# Heparan sulfate proteoglycans: a sugar code for vertebrate development?

**DOI:** 10.1242/dev.098178

**Published:** 2015-10-15

**Authors:** Fabienne E. Poulain, H. Joseph Yost

**Affiliations:** 1 Department of Biological Sciences, University of South Carolina, Columbia, SC 29208, USA; 2 University of Utah, Department of Neurobiology and Anatomy, Department of Pediatrics, Salt Lake City, UT 84132, USA

**Keywords:** Patterning, Left/right asymmetry, Nervous system, Heart, Glycan, Sugars

## Abstract

Heparan sulfate proteoglycans (HSPGs) have long been implicated in a wide range of cell-cell signaling and cell-matrix interactions, both *in vitro* and *in vivo* in invertebrate models. Although many of the genes that encode HSPG core proteins and the biosynthetic enzymes that generate and modify HSPG sugar chains have not yet been analyzed by genetics in vertebrates, recent studies have shown that HSPGs do indeed mediate a wide range of functions in early vertebrate development, for example during left-right patterning and in cardiovascular and neural development. Here, we provide a comprehensive overview of the various roles of HSPGs in these systems and explore the concept of an instructive heparan sulfate sugar code for modulating vertebrate development.

## Abstract (precis)

**Summary:** This Review article examines the role of heparan sulfate proteoglycans in vertebrate development and explores the concept of an instructive 'sugar code' for modulating development.

## Introduction

Vertebrate development – from a fertilized egg to a whole organism – is a complex process orchestrated by many signaling pathways. The generation of various cell types and organs from a single cell is achieved through successive developmental programs that require the spatial and temporal coordination of developmental signaling molecules, including morphogens and growth factors. Evidence accumulated over the past twenty years has shown that most, if not all, of these developmental factors are regulated by heparan sulfate proteoglycans (HSPGs).

**Fig. 1.**
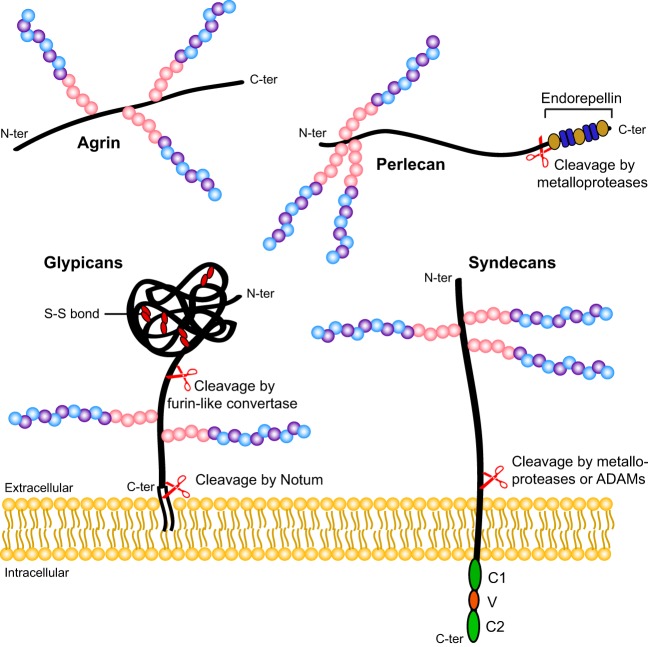
**Examples of key cell surface and extracellular HSPGs.** Glypicans (Gpcs) are attached to the cell surface via a glycosylphosphatidylinositol (GPI) anchor. They possess a large globular domain stabilized by conserved di-sulfide (S-S) bonds, and HS chains (represented by chains of pink, blue and purple circles) in their C-terminal part. They can be released into the extracellular matrix following cleavage of their GPI anchor by the lipase Notum. A furin-like convertase can also cleave Gpcs at the C-terminal end of their globular domain, leading to the formation of two subunits that remain attached to each other by disulfide bonds. Syndecans (Sdcs) are single-pass transmembrane proteins with HS chains attached to their N-terminal part. Their intracellular region interacts with many different partners through two conserved domains, constant 1 (C1) and constant 2 (C2), that are separated by a more variable region (V). Like Gpcs, Sdcs can be shed into the extracellular environment after cleavage by proteases such as matrix metalloproteases and a disintegrin and metalloproteinase (ADAM) disintegrins. Agrin and perlecan are large multidomain proteoglycans that carry several HS chains and are secreted as different isoforms generated by alternative splicing. The cleavage of the C-terminal region of perlecan by metalloproteases releases endorepellin, an angiogenesis inhibitor. C-ter, C-terminus; N-ter, N-terminus.

**Fig. 2.**
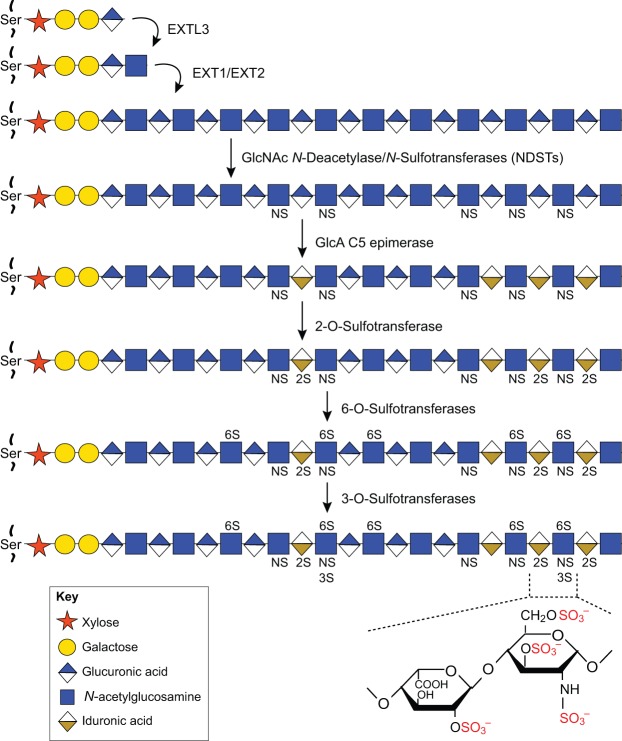
**The synthesis of heparan sulfate chains.** Heparan sulfate (HS) chains are linked by a xylose and a tetrasaccharide linkage to a specific serine residue of a core protein. This linkage region is identical in HS and chondroitin (CS) chains. The addition of the first *N*-acetylglucosamine is then catalyzed by the enzyme EXTL3. Elongation of the chains is achieved through the alternative addition of glucuronic acid and *N*-acetylglucosamine by the transferases EXT1 and EXT2. During polymerization, chains undergo several modifications including epimerization of glucoronic acid and sulfations at different positions. These modifications occur in different clusters and generate N-acetylated, N-sulfated and mixed domains differently involved in interactions with partners. Ser, serine; NS, N-sulfate group on glucosamine residues; 2S, 2-O-sulfate group on glucuronic or iduronic acid residues; 3S, 3-O-sulfate group on glucosamine residues; 6S, 6-O-sulfate group on glucosamine residues. Adapted from Esko et al., 2009.

HSPGs are composed of a core protein to which long linear glycosaminoglycan heparan sulfate (HS) chains are covalently linked. Syndecans (Sdc) and glypicans (Gpc), which are among the main classes of core protein, are anchored to the cell surface by a transmembrane domain or a glycosylphosphatidylinositol anchor, respectively, and can be released into the extracellular space following cleavage by various enzymes (Fig. 1). Other HSPGs, including agrin, collagen XVIII and perlecan, are directly secreted into the extracellular matrix (ECM). Core proteins have specific numbers of HS chain addition sites, and some also harbor chondroitin sulfate (CS) as another type of glycosaminoglycan (Table S1). HS chains are synthesized in the Golgi apparatus in a multi-step process that involves several enzymes (Fig. 2; Table S2). Exostosin (Ext) enzymes elongate HS chains by adding alternating glucuronic acid and *N*-acetylglucosamine residues, whereas N-deacetylase/N-sulfotransferase (Ndst) enzymes, C5 epimerase and 2-O-, 3-O-, and 6-O-sulfotransferases modify them by catalyzing deacetylation, epimerization and sulfations at different positions (for nomenclature, see Lawrence et al., 2008). These modifications do not occur uniformly along the HS chains but instead are concentrated in different sub-regions, creating domains with variably modified disaccharides that can interact with other proteins in distinct manners.

**Fig. 3.**
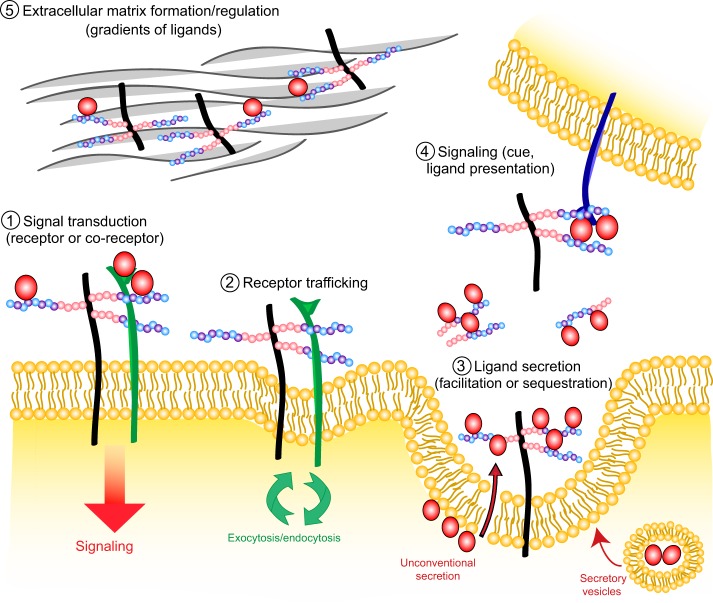
**The regulation of cell signaling pathways by HSPGs.** HSPGs regulate signaling pathways in many different ways, both at the intracellular level and in the extracellular matrix (ECM). HSPGs have been shown to: (1) mediate signal transduction by acting as receptors or co-receptors; (2) regulate receptor trafficking to and from the plasma membrane; (3) control the secretion of ligands; (4) signal to other cells by acting themselves as cues or presenting ligands to their receptors; and (5) regulate the structure of the ECM and the establishment of signaling gradients.

Given their various HS chains and localization (i.e. at the cell surface or in the ECM), HSPGs have been proposed to regulate signaling pathways in many different ways (Fig. 3). They can act cell-autonomously as receptors or co-receptors [for instance, during Wnt or fibroblast growth factor (FGF) signaling], as ‘recruiters’ to lipid rafts (thereby increasing ligand or receptor concentration at the cell surface), by regulating receptor membrane trafficking (during endocytosis) or by controlling ligand secretion. They can also act non-cell-autonomously as direct cues, or by controlling the distribution of signaling gradients as well as the composition of the ECM. Adding to the complexity of their signaling functions, HSPGs can interact with proteins through various mechanisms (for reviews, see Lindahl and Li, 2009; Xu and Esko, 2014). Binding to proteins is generally mediated by HS chains, but some factors such as Hedgehog (Hh) have been shown to directly interact with core proteins (Capurro et al., 2008). Whereas some interactions require specific structural motifs along the HS chains (Thunberg et al., 1982; de Paz et al., 2007), others are non-selective in terms of motifs but require a certain type of sulfation (for instance, 2-O-sulfation is essential for Fgf2 binding; Ashikari-Hada et al., 2004) or a certain negative charge density. Interestingly, it has been demonstrated that distinct organs or tissues produce unique compositions of HS, and that HS epitope expression changes during embryonic development in a tissue-specific manner (Allen and Rapraeger, 2003; Ledin et al., 2004). This observation, together with the large variability of HS chains that can be generated by the HS-modifying enzymes, has led to the hypothesis of a complex ‘sugar code’ (Holt and Dickson, 2005), in which specific HS modifications present in a region-specific manner in the embryo would orchestrate developmental programs by interacting with and modulating defined signaling pathways. This sugar code would thus depend on the spatial and temporal regulation of HS-modifying enzymes, and perhaps on different members of the same family of HS-modifying enzymes having slightly different activities or specificities of actions. For example, some family members of the 3-O-sulfotransferase family might have a greater or lesser propensity to make a modification adjacent to other sulfation marks, changing both the configuration of sulfation marks and the charge density in particular regions of an HS chain. The sugar code would also have to be dynamic, as HS chains can be degraded or modified at the cell surface by heparanase and sulfatase enzymes, respectively.

Although studies in *Caenorhabditis elegans* have strongly supported the sugar code hypothesis (Bülow et al., 2008; Tecle et al., 2013; Díaz-Balzac et al., 2014), evidence for specific roles of HS modifications and core proteins in vertebrates is only beginning to emerge. Nonetheless, accumulative data clearly indicate that HSPGs have distinct instructive roles during vertebrate development. For example, studies have shown that many organs, including the hematopoietic and musculoskeletal systems, and the liver, lungs and kidneys, do not form properly when HSPGs are absent or modified (Tables S1, S2; for reviews, see Thompson et al., 2010; Huegel et al., 2013; Nigam and Bush, 2014). These studies have utilized various model organisms, including mice, zebrafish and *Xenopus*, although it should be noted that some disparities between results have arisen, perhaps owing to the technical challenges of assessing HSPG functions *in vivo* (see Box 1). In this Review, we discuss the specific functions of HSPGs during vertebrate development, focusing on the early stages of development and the formation of the cardiovascular and nervous systems. We then evaluate the concept of the sugar code hypothesis, discussing evidence for and against it, and highlighting future challenges for the field.

**Box 1. Analyzing HSPG functions in development: approaches and challenges**

The functions of HSPGs in development have been assessed using various approaches, including targeted mutants in mice, genetic mutants in zebrafish and morpholino (MO) knockdowns in zebrafish and *Xenopus*. However, a growing challenge in the field is to reconcile the disparity among the phenotypes generated by these different methods (Tables S1, S2). In the absence of genetic mutants, the roles of HSPGs in zebrafish have mostly been assessed based on MO phenotypes, and these studies have provided some interesting insights into the specific roles of HSPG core proteins and biosynthetic enzymes in embryonic morphogenesis. There are, however, increasing concerns about potential MO artifacts and the possibility that mutants can be functionally compensated by other family members or other regulatory events (Kok et al., 2015; Stainier et al., 2015). As such, conclusions from MO-based studies await confirmation by engineered mutants in zebrafish. It is also somewhat surprising that mutants for some of the core protein genes, such as *sdc2* or *gpc5*, have not yet been reported in mice, and that mutations in the same gene (for instance *gpc4*) cause drastically different phenotypes in mice versus zebrafish or *Xenopus*. It is thus likely that a fuller understanding of HSPG functions will require the combination of multiple mutants, transgenic and other techniques to test lineage-specific roles of HSPG core proteins and biosynthetic pathways in vertebrate development.

## Roles for HSPGs in early development

**Fig. 4.**
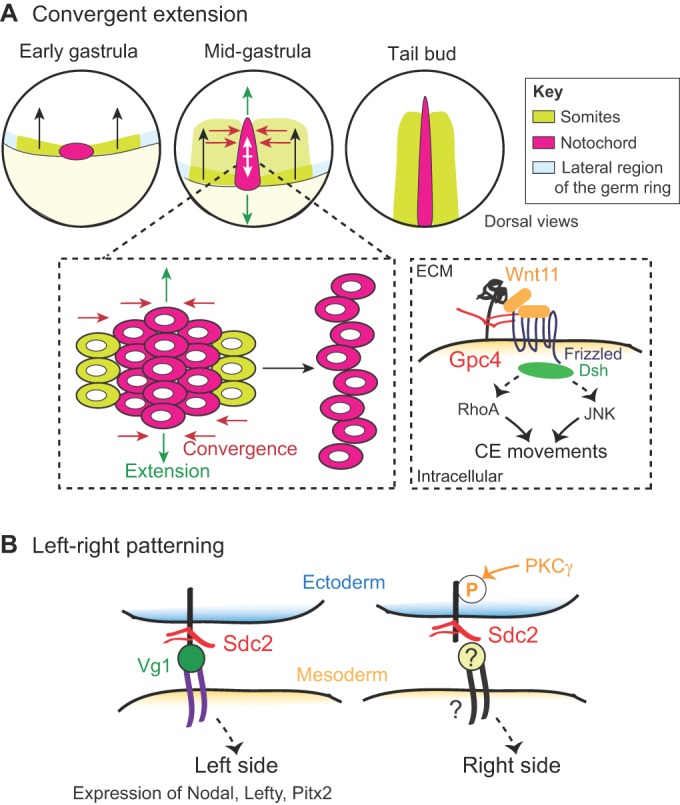
**Functions of HSPGs in early development.** (A) Convergent extension allows body axis elongation along the anteroposterior (AP) axis during gastrulation. The collective migration of lateral mesendoderm progenitors (presumptive somites, green) towards the midline (red arrows) and the cell intercalation (white arrows) of notochord progenitors (pink) lead to the extension of the notochord along the AP axis (green arrows). In zebrafish and *Xenopus*, Gpc4 regulates these convergent extension (CE) movements by modulating the Wnt11 pathway. (B) Normal left-right (LR) patterning in *Xenopus* requires both non-phosphorylated Sdc2 on the left and PKCγ-dependent phosphorylated Sdc2 on the right side of the embryo. Sdc2 signals non-cell-autonomously from the ectoderm, possibly by activating or mediating Vg1 signaling on the left side, which leads to asymmetric patterning of the mesoderm characterized by the expression of Nodal, Lefty and Pitx2 on the left side.

The first stages of development following egg fertilization (including cell cleavage, gastrulation and patterning) establish the different body axes and overall architecture of the embryo. Studies of mutants that are defective for genes encoding HSPG biosynthetic enzymes promote the view that HS sugar chains regulate multiple steps throughout these early developmental processes (Fig. 4A). HS chain polymerization has been shown to be crucial for patterning and early developmental function. Homozygous knockout (KO) mutants of *Ext1* or *Ext2* in mice are embryonic lethal, exhibiting defects in gastrulation (Lin et al., 2000; Mitchell et al., 2001; Stickens et al., 2005; Shimokawa et al., 2011). Zebrafish *ext2* (*dackel*) or *extl3* (*boxer*) mutants undergo normal gastrulation owing to a maternal contribution of both enzymes, but later develop cartilage and limb defects and display impaired FGF, Wnt and Shh signaling (Grandel et al., 2000; Norton et al., 2005; Clément et al., 2008; Holmborn et al., 2012). HS chain modifications also appear to be important, as the absence of both *Ndst1* and *Ndst2*, which encode enzymes that catalyze HS deacetylation and N-sulfation, is embryonic lethal in mice (Grobe et al., 2002), and epiboly is disrupted in zebrafish embryos injected with morpholinos (MOs) against *hs2st*, which encodes a sulfotransferase that adds sulfate to the C2 position of hexuronic acid residues along the HS chain (Cadwalader et al., 2012). Interestingly, the other reported mutants in HS-modifying enzymes only show later developmental defects (Table S2), suggesting functional redundancy between multiple gene family members that can contribute to specific biochemical steps in HSPG synthesis. Analyzing combinations of mutants will be required to uncover the early developmental functions of HSPGs.

Studies of mutants that are defective for individual core proteins also provide evidence for early developmental roles for HSPGs. In zebrafish, *gpc4* (*knypek*) mutants exhibit defects in Wnt11-dependent convergent-extension cell movements during gastrulation, suggesting that the potentiation of Wnt11 signaling is dependent on HSPG function (Topczewski et al., 2001). Gastrulation and posterior elongation defects are also observed in *Xenopus* embryos lacking or overexpressing *gpc4* (Ohkawara et al., 2003). By contrast, *Gpc4* KO mutants in mice develop normally and only show later defects in neural synapse formation (Allen et al., 2012). Other reported core proteins mutants have later onset developmental defects (Table S1), including a wide range of specific neurodevelopmental defects (discussed below), that often result in perinatal death.

## Roles for HSPGs in left-right patterning

Kupffer's vesicle (KV) is a ciliated organ that controls left-right (LR) patterning in the zebrafish embryo. During normal development, KV cells migrate and undergo a mesoderm-to-epithelial transition to form a vesicle. As the vesicle is inflated, each KV cell grows a specialized apical cilium that is motile and moves fluid asymmetrically in the KV, thereby establishing LR patterning throughout the embryo. A number of studies have implicated a role for HSPGs during this process of LR patterning (Fig. 4B).

The lineage-specific targeting of *sdc2* in zebrafish KV cells with MOs results in disrupted KV morphogenesis and shortened cilia, giving rise to altered LR patterning throughout the embryo (Arrington et al., 2013). Whole-embryo *sdc2* knockdown also causes later defects in vasculogenesis (Chen et al., 2004). Roles for HSPGs in LR patterning have also been assessed in *Xenopus* embryos, which offer the ability to identify and target cell lineages more precisely by microinjection. For example, the lineage-specific expression of dominant-negative mutant Sdc2 isoforms by targeted injection of mRNAs into individual *Xenopus* cells results in LR patterning defects (Kramer and Yost, 2002). Strikingly, the dominant-negative Sdc2 isoforms, including phosphomimetic and phosphodeficient forms that have altered phosphorylation sites in the cytoplasmic domain of the core protein, indicate that normal LR patterning requires both non-phosphorylated Sdc2 on the left and protein kinase C gamma (PKCγ)-dependent phosphorylated Sdc2 on the right side of the embryo (Kramer et al., 2002). This reveals a developmental role for asymmetric Sdc2 phosphorylation in LR patterning. However, it is possible that both the dominant-negative constructs and the MOs affect more than just Sdc2; dominant-negative Sdc2 might, for example, disrupt the complex formed by Sdc2 and Sdc4. It is therefore important to confirm these observations using combinations of mutants in the Sdc family.

HS chain modifications catalyzed by 3-O-sulfotransferases (encoded by Hs3st genes) have also been implicated in LR patterning. The MO-based lineage-specific targeting of *hs3st1l2* (also known as *hs3st5*) and *hs3st3l* (also known as *hs3st6*) in the zebrafish KV results in normal KV morphogenesis, but causes LR patterning defects that appear to be due to distinct perturbations in KV cilia length and cilia motility (Neugebauer et al., 2013). This study also implicated 3-O-sulfotransferase modifications in the regulation of Fgf8 and other cell signaling pathways. Few other mutants have been reported for the Hs3st family, although it should be noted that knockouts of *Hs3st1* in mice have subtle intrauterine growth retardation that is background dependent (Shworak et al., 2002; HajMohammadi et al., 2003).

## HSPG functions in cardiovascular development

Roles for core proteins as well as HS modifications in the development of the cardiovascular system have been identified. During early organogenesis, the mesoderm lineages that give rise to the heart and the mesoderm of the gut are present at the lateral edges of the vertebrate embryo and migrate towards the embryonic midline, fusing to form the cardiac and gut tube, respectively. The MO-mediated lineage-specific knockdown of zebrafish *sdc2* reveals a role for this HSPG core protein in the non-cell-autonomous regulation of ECM formation, which is required for cardiac and gut mesoderm migration. In particular, the disruption of *sdc2* function in embryonic yolk cells leads to altered ECM formation throughout the embryo and impedes the migration of both cardiac and gut precursor cells, resulting in cardia bifida (two separate heart tubes) and bifid gut tubes (Arrington and Yost, 2009). Among the other core proteins, only Gpc3 and Perlecan (Hspg2 – Zebrafish Information Network) have so far been implicated in heart development. Congenital cardiac malformation, including ventricular septal defects or double outlet right ventricle, have been reported in mice lacking *Gpc3* and seem to result from impaired Shh signaling in the heart (Ng et al., 2009). Perlecan-deficient mice show a different phenotype, characterized by malformations of the cardiac outflow tract (Costell et al., 2002).

An important insight into the role of HS chains in cardiovascular development came from the analysis of *Ndst1* KO mice, which show several heart defects reminiscent of those observed in the absence of *Gpc3* (Pan et al., 2014). FGF signaling is also strongly reduced in *Ndst1* KO mice, and impaired Fgf8 function appears to contribute to their phenotype. The conditional deletion of *Ndst1* in neural crest cells in a systemic *Ndst2*-deficient background further indicates an important function of HS in the neural crest cell lineage that migrates into the developing cardiac tube during normal cardiac development, and suggests at least partially compensatory roles among Ndst family members. The functions of the other HS chain modifications in cardiovascular development remain difficult to address. Mice lacking C5-epimerase or Hs2st die at birth and show severe developmental defects (Table S2). Furthermore, various phenotypes have been reported for mice lacking *Hs6st1* (Pratt et al., 2006; Habuchi et al., 2007; Izvolsky et al., 2008), whereas double *Hs6st1* and *Hs6st2* mutants have enhanced phenotypes and are embryonic lethal (Conway et al., 2011b), indicating some level of functional redundancy between the members of the 6-O-sulfotransferase family. Zebrafish embryos injected with MOs against the 3-O-sulfotransferase *hs3st7* (*hs3st1l1*) exhibit normal LR patterning and early development but show defects in contraction of the cardiac ventricle. This myocardium contraction defect can be rescued by the transgenic expression of *hs3st7* in the endocardium tissue that lines the cardiac tube, but not in the myocardium, indicating that the regulation of myocardium contraction by *hs3st7* is non-cell-autonomous, implicating cell-cell signaling. Strikingly, genetic reduction of bone morphogenetic protein (BMP) signaling in the heart also rescues contraction in *hs3st7* morphants (Samson et al., 2013). Together, these results suggest that different members of the Hs3st family control distinct steps, and perhaps distinct cell signaling pathways, during cardiac development.

## Roles for HSPGs during nervous system development

**Fig. 5.**
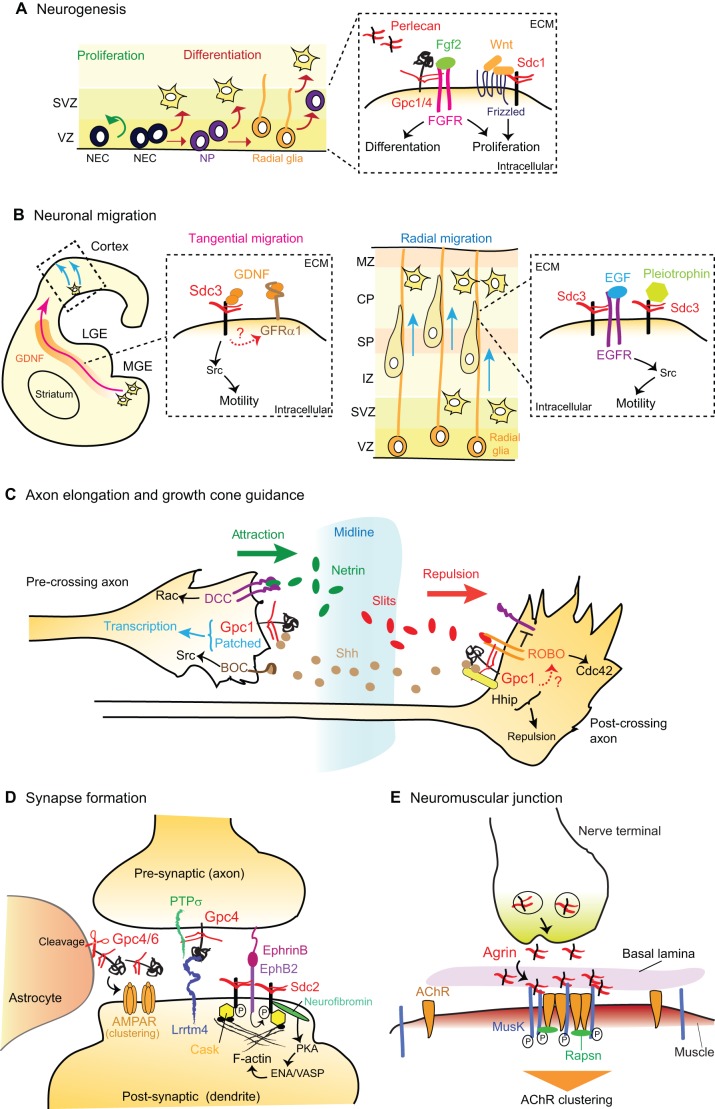
**Functions of HSPGs in nervous system development.** (A) During neurogenesis, neuroepithelial cells (NECs) act as neural stem cells (NSCs) that proliferate and differentiate in the ventricular and subventricular zones (VZ and SVZ) of the cortex, giving rise to neuronal progenitors (NPs), neurons and radial glia. Several factors required for neurogenesis are regulated by HSPGs: the Fgf2 pathway is controlled by Gpc1 and Gpc4; Sdc1 modulates the canonical Wnt pathway. Perlecan also acts in the extracellular matrix (ECM) to regulate NEC proliferation. (B) During neuronal migration in the cortex, Sdc3 regulates cell migration along radial glia (blue arrows), from the intermediate zone (IZ) to the marginal zone (MZ), by mediating pleiotrophin and EGFR signaling. Sdc3 also acts as a receptor for GDNF and mediates the tangential migration of inhibitory neurons along a GDNF gradient present from the medial ganglionic eminence (MGE) towards the cortex (pink arrow). CP, cortical plate; LGE, lateral ganglionic eminence; SP, subplate. (C) During axon elongation, guidance cues present at the midline, such as Netrin, Slits or Shh, require HSPGs for signaling through their respective receptors DCC, Robo and Patched, both *in vitro* and *in vivo* in *Drosophila* and *C. elegans*. *In vivo* in vertebrates, Gpc1 mediates the response of axons to Shh: in pre-crossing axons, Gpc1 interacts with Shh bound to Patched to promote the transcription of specific genes, including that of the Shh receptor Hhip. In post-crossing axons, Gpc1 binds to Shh, triggering a repulsive response through Hhip signaling. (D) During synaptogenesis, pre-synaptic Gpcs, in particular Gpc4, interact with the pre-synaptic receptor PTPσ and the post-synaptic protein Lrrtm4 to promote excitatory synapse development. Gpc4 and Gpc6 are also released from astrocytes and promote synapse formation by clustering post-synaptic AMPA glutamate receptors (AMPARs). On the post-synaptic membrane, EphB2 phosphorylates Sdc2 and induces its clustering. The interaction of Sdc2 with adaptor proteins, such as Cask or neurofibromin, promotes the formation of dendritic spines. (E) At the neuromuscular junction, agrin is released from the nerve terminal and becomes stabilized in the basal lamina. It binds and activates the receptor Musk, leading to AChR clustering via the cytoplasmic adaptor protein Rapsn.

Nervous system development proceeds through several partially concomitant steps that are regulated by a large diversity of environmental factors, such as morphogens, guidance cues, growth factors, adhesion molecules and components of the ECM, distribution and/or function of which are in large part controlled by HSPGs (Fig. 3). Although the crucial role of HS synthesis in nervous system formation was demonstrated over ten years ago, novel functions for HS modifications as well as core proteins are being discovered, suggesting that the diversity of these molecules might provide a ‘code’ regulating each aspect of nervous system development in an instructive manner (Fig. 5).

### Nervous system patterning

During its early development, the neural tube becomes regionalized into distinct domains that give rise to the major structures of the central nervous system. The crucial role of HSPGs in brain patterning was first demonstrated in *Nestin-cre; Ext1* conditional knockout (cKO) mice (*Nestin-cre*; *Ext1^flox/flox^*), in which HS synthesis is abolished in neural stem cells (NSCs) and, consequently, in the three lineages originating from them: neurons, astrocytes and oligodendrocytes (Inatani et al., 2003). These mice die at birth and show several patterning defects, including a lack of discrete cerebellum and the absence of olfactory bulbs. Interestingly, a similar phenotype is observed in hypomorphic *Fgf8* mutants (Meyers et al., 1998) and in the *swaying* mice, which exhibit a recessive mutation in *Wnt1* (Thomas et al., 1991). The analysis of Fgf8 immunoreactivity and of the expression domains of genes downstream of FGF in *Nestin-cre*; *Ext1^flox/flox^* mice revealed an expanded distribution and activity of Fgf8 in the absence of HS, suggesting that HSPGs regulate midbrain/hindbrain patterning by controlling Fgf8 levels in the environment. Supporting this hypothesis, a more recent study showed that the formation of an FGF posterior-to-anterior gradient along the midbrain is impaired after treatment with heparitinase or heparin (Chen et al., 2009).

Further investigations revealed that HS modifications appear to be essential for brain patterning. Mice lacking the N-deacetylase/N-sulfotransferase Ndst1 display forebrain defects, including cerebral hypoplasia and a lack of olfactory bulbs (Grobe et al., 2005). The regulated removal of 6-O moieties from HS chains at the cell surface also appears important, as mice lacking the endosulfatase Sulf2 show brain abnormalities, including neural tube closure defects, an enlargement of the ventricular system and hydrocephalus (although with partial penetrance) (Kalus et al., 2009).

More recently, specific core proteins involved in nervous system patterning have been identified. For example, mice lacking Gpc1 do not form the most anterior lobe of the cerebellum, a defect possibly related to the cerebellar agenesis seen in the *Nestin-cre; Ext1* cKO mice (Jen et al., 2009). The knockdown of *gpc4* in *Xenopus* leads to a different phenotype characterized by dorsal forebrain defects and an absence of anterior neural tube closure due to impaired FGF signaling (Galli et al., 2003). Defective neural tube closure is also observed in *Xenopus sdc4* morphants, and genetic interaction studies suggest that *sdc4* and *vangl2* act in the Wnt/PCP pathway to regulate this process (Muñoz and Larraín, 2006; Escobedo et al., 2013). Sdc4 has also been shown to activate the FGF/ERK and PKC signaling pathways to trigger neural induction (Kuriyama and Mayor, 2009).

### Neurogenesis

Neurogenesis – the generation and differentiation of neurons from NSCs – occurs predominantly during development, but also continues in the adult in specific regions of the brain, such as the olfactory bulb, the rostral migratory stream, the subgranular zone in the dentate gyrus of the hippocampus, or the subventricular zone (SVZ) lining the ventricles. Neurogenesis is regulated by several morphogens and growth factors, including Fgf2, which plays a dominant role (Guillemot and Zimmer, 2011) and requires HS for its activity. Accordingly, the proliferation of cultured cortical progenitors from *Nestin-cre; Ext1* cKO mice in response to Fgf2 is dramatically reduced compared with that of progenitors from wild-type mice (Inatani et al., 2003). Consequently, these mutants show impaired neurogenesis leading to a smaller forebrain and a strongly reduced cortical thickness. Interestingly, a similar phenotype is observed in the absence of Hs2st, suggesting an important role for 2-O-sulfation in NSC proliferation (McLaughlin et al., 2003; Pratt et al., 2006).

Specific core proteins also play major roles in neurogenesis. The expression of perlecan is increased in response to Fgf2 (Mashayekhi et al., 2011), and mice lacking perlecan (Hspg2 – Mouse Genome Informatics) show reduced cell proliferation leading to microcephaly with thinner cerebral walls (Girós et al., 2007). Perlecan is also required for the maintenance of NSCs, as both the size of the NSC population and the extent of neurogenesis are strongly reduced in the SVZ of adult mice lacking perlecan (Kerever et al., 2014). Among membrane-associated HSPGs, Gpc1 appears to contribute significantly to the regulation of neurogenesis. Mice lacking *Gpc1* exhibit a reduced brain size as a result of decreased proliferation (by 20%) from embryonic day (E) 8.5 to E9.5 (Jen et al., 2009). This modest but significant effect seems to be mediated by a reduction in Fgf17 signaling, and might be partly compensated by Gpc4. The analysis of compound mutants (*Gpc1* and a gene-trap allele of *Gpc4*) does indeed suggest that Gpc1 and Gpc4 might have overlapping functions in regulating brain size (Jen et al., 2009). Gpc4 is strongly expressed in the ventricular zone of the telencephalon as well as in neurogenic regions in adult mice. Its ability to bind Fgf2 further suggests a possible role for Gpc4 as a modulator of FGF signaling during neurogenesis (Hagihara et al., 2000). Additional studies will be required to test the contribution of Gpc4, as the gene-trap allele of *Gpc4* is thought not to be completely null. Finally, a recent study has highlighted a novel function for Sdc1 in neurogenesis (Wang et al., 2012). *Sdc1* is highly expressed in progenitors in the ventricular zone and SVZ during development. Inhibiting its expression by electroporating a short hairpin RNA *in utero* reduces the proliferation of NPCs in response to Wnt and leads to premature differentiation. Interestingly, Sdc1 modulates the canonical Wnt pathway but does not appear to regulate the FGF effector Erk1/2 (Mapk3/Mapk1) (Wang et al., 2012). This is in stark contrast to the mode of action employed by Gpc1 (Jen et al., 2009), supporting the hypothesis that the diversity of HSPGs might fine-tune the regulation of the different pathways orchestrating nervous system development.

### Neuronal migration

After neurogenesis and the establishment of neuronal identity, individual post-mitotic neurons migrate to their final functional destination. Several types of migration occurring in different waves have been described. Some involve the movement of neurons along radial glial fibers, such as the radial migration of newborn neurons from the ventricular zone to form the different layers of the cortex (Nadarajah and Parnavelas, 2002). In others (for instance, the migration of neurons into the olfactory bulb), neurons do not use glia as a scaffold and instead migrate ‘in chain’ (Lois et al., 1996). HSPGs appear to be involved in regulating both of these types of migration. Sdc3 in particular appears to play an essential role in neuronal migration in mammals (Hienola et al., 2006; Bespalov et al., 2011). Mice lacking Sdc3 have a disorganized laminar structure of their cortex owing to impaired radial migration along glia. ‘In chain’ migration along the rostral migratory stream towards the olfactory bulb is also abnormal. Both defects seem to arise from a defective response of neural cells to the migration-promoting factors pleiotrophin and epidermal growth factor (EGF). Accordingly, it was shown that the Sdc3 ectodomain binds to pleiotrophin and the epidermal growth factor receptor (EGFR), and that forebrain cells isolated from *Sdc3* KO mice show a reduced migration response to EGF and pleiotrophin *in vitro* (Hienola et al., 2006). Whether Sdc3 requires its HS chains to mediate pleiotrophin and EGF signaling remains to be determined. More recently, Sdc3 has been identified as a novel receptor for glial cell-derived neurotrophic factor (GDNF) (Bespalov et al., 2011). GDNF binds to Sdc3 in a HS-dependent manner and requires Sdc3 to promote the migration of cortical neurons *in vitro*. This observation translates *in vivo*: cortical GABAergic neurons known to migrate along a GDNF gradient from the medial ganglionic eminence towards the cortex (Pozas and Ibáñez, 2005) do not migrate properly in mice lacking *Sdc3* (Bespalov et al., 2011).

Surprisingly, although the inhibition of global HS synthesis in *C. elegans* by RNAi against *rib-1* or *rib-2* (homologs of the mammalian Ext genes) disrupts neuronal migration (Pedersen et al., 2013), no obvious defects in cortical lamination patterns or localization of specific neuronal populations [calbindin 2 (also known as calretinin)-positive and Pou3f1 (also known as Tst-1 and SCIP)-positive neurons] could be observed in *Nestin-cre; Ext1* cKO mice (Inatani et al., 2003). This suggests that the effects of HSPGs on neuronal migration may be species specific, or that compensatory mechanisms may exist in mice.

### Axon growth and guidance

Concomitantly or subsequent to their migration, neurons extend axonal processes towards their future synaptic partners. During its navigation, the axon is guided by a plethora of attractive and repulsive guidance cues that act via direct contact or over a distance in the environment. A crucial role for HS during axon pathfinding has been known for a long time. Major guidance cues, such as Netrins, Slits, and Ephrins, have been shown to bind HS with a high affinity (Serafini et al., 1994; Bennett et al., 1997; Hussain et al., 2006; Irie et al., 2008). Furthermore, adding HS to the developing *Xenopus* retinotectal pathway or removing HS with heparitinase prevents retinal axons from entering their brain target, the tectum (Walz et al., 1997; Irie et al., 2002). Not surprisingly, *Nestin-cre; Ext1* cKO mice lacking HS in the nervous system show major axon guidance defects, including an absence of commissural tracts and errors from retinal axons (Inatani et al., 2003). Similarly, retinal axons erroneously project into the forebrain, the hindbrain and the opposite eye in *ext2; extl3* zebrafish mutants, which also exhibit decreased HS levels (Lee et al., 2004). Mis-sorting of retinal axons along the optic tract is also observed in these mutants and has revealed an important function of HS in developmental axon degeneration (Poulain and Chien, 2013). Indeed, the topographic organization of retinal axons along the tract is achieved through the selective degeneration of mis-sorted axons in wild-type animals. This correction mechanism requires the presence of HS in the environment and is impaired in *ext2* mutants.

The first observation that different HS-modifying enzymes regulate distinct aspects of axon navigation came from elegant work in *C. elegans* that built the foundation for the hypothesis of a ‘sugar code’ (Bülow and Hobert, 2004; Bülow et al., 2008). *C. elegans* has single orthologs of the various HS-modifying enzymes, making genetic manipulation and analysis of the resulting phenotype easier: deacetylation and N-sulfation are catalyzed by HST-1, epimerization is mediated by HSE-5, and 2-O, 3-O and 6-O sulfations are performed by HST-2, HST-3.1 and HST-3.2, and HST-6. Interestingly, different classes of neurons require the activity of HSE-5, HST-2 and HST-6 in distinct combinations for their axons to be correctly guided. HST-3.1 and HST-3.2 regulate more refined steps of morphogenesis, controlling branching in a context-dependent manner (Tecle et al., 2013; Gysi et al., 2013). In mammals, however, the contribution of specific HS motifs to axon guidance is only beginning to emerge. Like *Nestin-cre; Ext1* cKO mice, mouse mutants lacking Ndst1 show absent or hypoplastic anterior and hippocampal commissures (Grobe et al., 2005). Supporting the sugar code hypothesis, mouse retinal axons lacking Hs2st or Hs6st1 make distinct errors at the chiasm resulting from impaired responses to Slit1 and Slit2 (Pratt et al., 2006; Conway et al., 2011a; Conway et al., 2011b). Similarly, callosal axon navigation at the midline is differently impaired in the absence of Hs2st or Hs6st1 (Conway et al., 2011a; Conway et al., 2011b; Clegg et al., 2014). Interestingly, Fgf8 expression is increased and leads to a strong activation of ERK in the telencephalon of *Hs6st1* KO mice, but not in *Hs2st* mutants. In addition, suppressing components of the Fgf8/Erk pathway improves the phenotype of mice lacking Hs6st1, suggesting that Hs2st and Hs6st1 might regulate callosal axon guidance in a molecularly distinct manner.

Although the phenotypes observed in the absence of HS strongly suggest a role for HSPGs in axon pathfinding, very little is known about the roles of the HSPG core proteins. Most studies describing functions for specific core proteins in axon guidance have been performed in *Drosophila* and *C. elegans*, in which one Sdc and two Gpc genes have been identified. For example, it was shown in both organisms that mutations in *Sdc* induce defects in midline axon guidance through impaired Slit/Robo signaling (Johnson et al., 2004; Steigemann et al., 2004; Rhiner et al., 2005; Smart et al., 2011). Both Sdc and the Gpc Dally-like are required for proper axon pathfinding and visual system function in *Drosophila* (Rawson et al., 2005). Finally, the Gpc *lon-2* regulates motor axon guidance in *C. elegans* (Bülow et al., 2008). In vertebrates, only one recent study has demonstrated a role for Gpc1 in mediating the repulsive response of postcrossing axons to Shh at the floorplate in chick (Wilson and Stoeckli, 2013).

### Dendritogenesis

As axon outgrowth proceeds, neurons develop branched dendritic arbors that are responsible for receiving, integrating and propagating signals received from other neurons. The resulting dendritic morphology is unique to each specific neuronal class and is thought to be determined by both environmental and cell-intrinsic factors. Sdc2 has been shown to regulate dendrite formation in hippocampal neurons in culture by interacting with the multidomain adaptor molecule Sarm1 and activating the JNK/MKK4 pathway (Chen et al., 2011). It would be interesting to generate *Sdc2* KO mice and test whether Sdc2 has a similar role *in vivo*. Very little is known about the role of other HSPG core proteins or HS-synthesizing enzymes in dendritogenesis, and further studies are required to address their functions.

### Synaptogenesis

Synapses are specialized adhesive junctions that enable the transmission of information between neurons (in the central nervous system) or between neurons and muscles (at neuromuscular junctions, NMJs). Neurotransmitters are released from presynaptic axonal termini into the synaptic cleft and bind to their receptors on the postsynaptic membranes of dendrites or muscles fibers. In the central nervous system, post-synaptic membranes are organized on dendrites into small protrusions of various shapes called spines. These spines evolve from actin-rich thin filopodial protrusions and develop into mushroom-like or stubby shapes during their formation and maturation.

The role of HSPGs in synaptogenesis was first demonstrated at the NMJ nearly 20 years ago. This early demonstration showed that the HSPG agrin, named after its ability to induce the aggregation of nicotinic acetylcholine receptors (AChRs) on developing skeletal muscle fibers in culture (Godfrey et al., 1984), is secreted from the terminal end of motor axons and initiates the formation of neuromuscular synapses by binding to and activating the receptor tyrosine kinase Musk on the muscle surface (Glass et al., 1996). Mice lacking agrin do not form NMJs and die *in utero* (Gautam et al., 1996). Similarly, a homozygous missense mutation in the agrin gene has been identified in a case of congenital myasthenic syndrome in humans, in which abnormal neuromuscular transmission leads to muscle weakness accentuated by exertion (Huzé et al., 2009). In addition to its high expression levels in motor neurons, agrin is detected in the central nervous system, and it was shown that blocking agrin function in cultured hippocampal neurons leads to the abnormal formation of synapses (Ferreira, 1999; Bose et al., 2000). Synapse loss has also recently been described in the cortex of transgenic adult mice lacking agrin expression everywhere but in motor neurons, confirming a role for agrin in the formation and/or maintenance of synapses in the brain (Ksiazek et al., 2007).

Studies of cultured hippocampal neurons have also revealed a role for Sdc2 in the formation of dendritic spines. Such a role had been suggested by the high expression levels of Sdc2 during synaptogenesis in the nervous system, and the identification of the synaptic protein Cask (calcium/CaM-dependent serine protein kinase) as an Sdc2-interacting partner (Cohen et al., 1998; Hsueh et al., 1998). Overexpressing *Sdc2* in rat hippocampal neurons accelerates spine formation, whereas inhibiting its expression prevents the development of dendritic protrusions (Ethell and Yamaguchi, 1999; Lin et al., 2007). The ability of Sdc2 to promote filopodia formation and synaptogenesis requires different domains in its intracellular region (Fig. 1). The C1 domain interacts with neurofibromin, activates PKA and promotes ENA/VASP activity underlying filopodia formation (Lin et al., 2007). Phosphorylation of the C1 domain by the EphB2 receptor tyrosine kinase has also been shown to be required for Sdc2 clustering on dendrites and the induction of dendritic spines (Ethell et al., 2001). The C2 region, which contains a type II PDZ-binding motif (residues EFYA) that mediates interactions with several adaptor proteins such as syntenin (Sdcbp), Cask, synbindin (Trappc4) and synectin (Gipc1), is also required for Sdc2 function in spines. However, the contribution of HS chains to Sdc2 function, as well as the role of Sdc2 *in vivo*, remains unknown.

In addition to Sdc2, some Gpcs have recently been implicated in the regulation of synaptogenesis. Two independent studies in rodents and *in vitro* identified Gpcs (most notably Gpc4) as pre-synaptic partners of the leucine-rich repeat transmembrane neuronal protein Lrrtm4, which is located at the post-synaptic membrane (de Wit et al., 2013; Siddiqui et al., 2013). This trans-synaptic interaction involves the HS-dependent binding of Gpc4 to the pre-synaptic receptor protein tyrosine phosphatase PTPσ (Ko et al., 2015) and is required for Lrrtm4 to promote excitatory synapse development. Additionally, Gpc4 and Gpc6 have been shown to be secreted from astrocytes and to promote excitatory synapse formation in retinal ganglion cells through clustering of glutamate receptors (Allen et al., 2012). Generating and analyzing mutants for the different Gpc genes will be of high interest to validate these exciting results *in vivo*.

Although the functions of the various core proteins in synaptogenesis are emerging, at least *in vitro*, the roles of HS chain modifications remain poorly characterized. HS is enriched in synapses of adult neurons, and cKO mice lacking *Ext1* in post-mitotic neurons exhibit altered glutamatergic synaptic transmission and develop numerous autism-like behavioral deficits (Irie et al., 2012). Mental impairment and autism have similarly been described in patients with deletion mutations in *EXT1* (Li et al., 2002), demonstrating a role for HS in synaptic and cognitive function. Interestingly, a recent screen performed in *Drosophila* showed different effects of *Sulf1* and *Hs6st* on synapse formation and function at the NMJ (Dani et al., 2012): mutants lacking Sulf1 or Hs6st both have an increased number of synaptic boutons but show striking opposite differences in their neurotransmission strength. Sulf1 and Hs6st thus appear to share a similar function in synapse morphogenesis, but play opposing roles in synaptic functional differentiation. In addition, the cell surface expression of the Gpc Dlp is reduced in synapses lacking Hs6st, whereas both Sdc and Dlp are increased in the absence of Sulf1. These changes correlate with differences in Wnt anterograde and BMP retrograde trans-synaptic signaling, both of which are responsible for the pre- and postsynaptic molecular differentiation defects observed in mutants. The absence of *Sulf1* in mammals also appears to disturb synaptogenesis (Kalus et al., 2009), as hippocampal neurons lacking Sulf1 display a decreased density of dendritic spines and an altered synapse structure with a reduced number of synaptic vesicles. These defects lead to an impaired synaptic plasticity in adult *Sulf1* KO mice.

## Future directions and implications for the sugar code hypothesis

HSPGs have been shown in cell culture studies to regulate a wide range of cell signaling pathways, cell-cell interactions, extracellular matrix formation, and cellular behaviors implicated in developmental mechanisms. Most recent studies have confirmed that HSPGs do indeed play major roles, many of them developmental, *in vivo*. However, there remain a number of major challenges in the field.

One key aim is to use genetics, transgenics and other functional assays to discern the roles of HSPG core proteins and the biosynthetic pathways that generate modified sugar chains. Although mutant analyses of some of the genes have yielded exciting results, most genes (including most of the multigene Hs3st family) have not yet been tested (Tables S1, S2). Genetic functional analysis is likely to be complicated by partially overlapping functional redundancies, for example within the same biosynthetic enzyme family, between some members of the same core protein family, and perhaps between different core families. Although it is clear that some core proteins have distinct functions (Table S1), those functions could be independent of HS attachments to that core protein. Conversely, one could speculate that an Sdc core protein and Gpc core protein might be able to perform some of the same functions as long as they can acquire the same modified sugar chains (sugar code) and deliver that information to the cell surface in a particular cell lineage during development. Similarly, it might be possible for multiple members of the same biosynthetic gene family, for example the seven or eight members of the Hs3st family in vertebrates, to participate in generating the same sugar code in a particular cell lineage. Thus, it might be necessary to generate double mutants and beyond, as has been done for a few biosynthetic enzyme genes (Table S2). Despite these challenges, genetic analysis will be essential for testing the functions of HSPGs in development.

It should also be noted that the sugar code hypothesis is exciting but controversial. A number of key findings are in support of the hypothesis: distinct cells can generate unique compositions of HS; distinct HS structural motifs are associated with defined developmental events, especially in invertebrates; and some HS-protein interactions show specificity and selectivity. Of particular interest, it has been shown that 3-O-sulfation enzymes contribute to a very small level of HS modification and yet are represented by the largest gene family in the pathway (Thacker et al., 2014), suggesting that individual family members have branched out during evolution to perform slightly different functions. The notion of a family of enzymes having distinct functions in the generation of a sugar code is supported by biochemical evidence for differences in substrate preference within the same family of enzymes (Moon et al., 2012). However, a number of arguments against the sugar code have also been put forward. For example, the genetic absence of certain individual enzymes/core proteins does not trigger major defects, perhaps owing to functional redundancy or compensatory mechanisms, as seen in the comparison of single versus double mutants, or perhaps because the loss of one enzyme can also induce global changes in HS chain contents, which might provide some functional compensation. It has also been noted, *in vitro*, that some HS-protein interactions are relatively non-selective, as long as a specific type of sulfation (Ashikari-Hada et al., 2004) or an overall density of negative charges is provided by HS.

When considering genetic functional analyses of individual enzyme family members, it should also be noted that a phenotype could arise not because other enzymes in the same family cannot compensate for function, but because those enzymes are not expressed in the affected tissue. One of the great challenges is that, in contrast to RNA synthesis, HS synthesis is not template driven. Thus, if there is a sugar code, building it is likely to be dependent on the innate properties of the biosynthetic enzymes and their complexes, which are perhaps dependent on subtle substrate specificities as they decide where to add modifications to HS chains in the context of other modifications on that chain.

Finally, although powerful methods have been developed to study HS-protein interactions *in vitro* (Powell et al., 2004; Shipp and Hsieh-Wilson, 2007), with new technologies rapidly evolving, determining HS structure in biochemical and functional analyses in the context of *in vivo* development remains a real challenge. In analogy to advances in RNA expression profiling, one would ideally like to be able to analyze the ‘sequence’ of HS modifications, from one end of individual chains to the other, and to do so from a very heterogeneous mix of HS chains derived from single cells obtained from developing organisms. With these many challenges, there is clearly much work to be done to understand the roles of heparan sulfate proteoglycans in development.
